# Post-anthesis supplementary irrigation improves grain yield and nutritional quality of drip-irrigated rice (*Oryza sativa* L.)

**DOI:** 10.3389/fpls.2023.1126278

**Published:** 2023-04-05

**Authors:** Xiangbin Wang, Xinjiang Zhang, Linghui Liu, Xiaowu Liu, Guorui Feng, Juan Wang, Yong-an Yin, Changzhou Wei

**Affiliations:** ^1^ College of Agriculture, Shihezi University, Shihezi, Xinjiang, China; ^2^ Technical Center of Xinjiang Tianye (Group) Co., Ltd., Shihezi, Xinjiang, China

**Keywords:** water-saving cultivation, water productivity, source-sink relationship, starch synthesis, protein components

## Abstract

**Introduction:**

Approximately 50% of irrigation water is saved during drip-irrigation of rice, which has tremendous potential for water-saving agriculture, particularly in areas where water resources are scarce. However, the grain yield and quality of drip-irrigated rice are adversely affected.

**Methods:**

In this study, we investigated the effects of different irrigation strategies on the grain yield and quality of drip-irrigated rice using field experiments. Four irrigation treatments were studied: whole growing season flooding (FI), whole growing season normal drip irrigation (DI, soil relative moisture (RSM) was maintained in the range of 90-100%), pre-anthesis drip irrigation and post-anthesis water stress (SAF, the RSM was maintained in the range of 80-90% after anthesis), pre-anthesis drip irrigation, and post-anthesis flooding (FAF).

**Results:**

The results showed that grain yield, harvest index, seed setting rate and 1000 grain weight in DI and SAF were significantly lower than in FI and FAF. These parameters were not significantly different between FI and FAF but were significantly greater in DI than in SAF. Compared with FI and FAF, the source capacity, source activity time, and sink activity of DI and SAF decreased, and the sink-source difference increased. The sink-source difference had a significant negative correlation with rice yield and 1000 grain weight. The activities of ADP-glucose pyrophosphorylase, starch branching enzyme, and amylopectin content in grains in the middle panicles of FAF were significantly higher than those of DI and SAF. SAF resulted in increased amylose/amylopectin ratio and total protein content in grains but decreased proportion of glutenin in total protein. Irrigation after anthesis of drip-irrigated rice narrowed the difference between sink sources in rice plants, increased the grain yield and harvest index by 29.2% and 11%, respectively, compared to DI, increased water productivity by 19% compared to FI, and improved the grain quality of drip-irrigated rice.

**Discussion:**

This study highlights that post-anthesis sufficient irrigation of drip-irrigated rice plays a positive role in maintaining the source-sink balance. This study serves as a foundation for the development of more effective rice farming methods that conserve water, while increasing the grain yield and quality of drip-irrigated rice.

## Introduction

Rice (*Oryza sativa* L.) is one of the most water-dependent crops worldwide, and the amount of water required for its irrigation accounts for more than 43% of total agricultural water use (Humphreys et al., 2010; Lee et al., 2016). Water resources for use in irrigation are becoming increasingly scarce owing to increased population, urbanization, industrialization, climate change, and environmental degradation, which pose a severe threat to rice production and development (Peng et al., 2009; Chu et al., 2018). To address these global water issues, it is crucial to increase water productivity and conserve water resources through water control. In addition to genetic factors, environmental conditions such as temperature, atmospheric humidity, and soil moisture affect the grain yield and quality of rice. In the water-scarce Xinjiang province of China, the rice drip irrigation cultivation technique has been steadily developed and is regarded as crucial agronomic technology to address food problems and conserve water in dry regions. Drip irrigation consumes > 40% less water than flooded irrigation. (Sharda et al., 2016). Maintaining or even enhancing the yield and quality of drip-irrigated rice is a prerequisite for the advancement of drip irrigation cultivation technology, which is important long-term for the development of sustainable agriculture.

To date, most published studies on water regulation to improve rice yield and quality (Belder et al., 2004; Arai et al., 2021; Hong Trang et al., 2022) have focused on upland rice cultivation or alternating wet and dry irrigation, whereas the mechanisms underlying quality changes in rice under water-saving irrigation conditions have received less attention. According to several studies, moderate alternating wet and dry irrigation increases rice yield (Li et al., 2017) and promotes the filling of inferior grains by increasing the activity of key enzymes involved in starch synthesis (Chen et al., 2015). According to some studies, upland rice cultivation or alternating dry and wet cultivation enhances the sensory and nutritional qualities of rice. (Norton et al., 2017; Zhang et al., 2020; Song et al., 2021). However, some researchers have found that water-saving irrigation increases the chalkiness degree and amylose content of grains, thereby negatively affecting rice quality (Graham-Acquaah et al., 2019). In practice, it was found that the yield and quality of rice following drip irrigation were reduced, which may have been caused by slight water stress under drip irrigation (He et al., 2016; Zhang et al., 2019). In brief, current research tends to support the remobilization of assimilates under adequate drought stress to increase rice yield and quality (Suralta et al., 2018), although the majority of the research only focuses on alternate dry and wet irrigation. The rice drip irrigation system differs significantly from other water-saving irrigation methods such as alternating wet and dry irrigation systems. Alternating wet and dry irrigation is characterized by large changes in field water content (from flooding to drying and then to flooding), whereas drip irrigation is characterized by a small amount of high-frequency irrigation, which allows the field soil to maintain a higher water content with reduced formation of a water layer, causing rice, an aquatic plant, to suffer from mild water stress (Zhang et al., 2019). This leads to a decrease in rice yield and quality under drip irrigation in practice (He et al., 2016; Zhang et al., 2019). Therefore, enhancing rice quality and yield in drip irrigation systems still requires more research, which is crucial for the advancement of drip-irrigation rice cultivation.

Rice yield and quality are closely related to the source-sink relationship. Some studies have reported that organic substitution or N regulation can improve the balance between the source and sink to increase rice yield (Shi et al., 2017; Pan et al., 2022). Other studies showed that rehydration after drought can restore the metabolism of rice source and sink organs, thus ensuring the normal growth of rice (Lawas et al., 2019), but this irrigation method is still quite different from drip irrigation, and relevant knowledge still needs further study. Fu et al. (2011) used sucrose synthase and ADP-glucose pyrophosphorylase as indicators of sink activity; however, these starch synthetases are also closely related to the synthesis of amylose and amylopectin (Nakamura et al., 1989; Nakamura and Yuki, 1992). Therefore, the source-sink relationship should be examined to elucidate the process underlying rice yield and quality under drip irrigation.

We hypothesized that the period from anthesis to maturity is the optimal time to alter the yield and quality potential of rice for the significant water-saving irrigation method, drip irrigation, because the rice grain-filling process is closely related to grain yield and quality. Based on these findings, we conducted field trials using four irrigation treatment strategies. The objectives of this study were to (1) clarify the mechanism underlying the reduction of grain yield and quality by drip irrigation, and (2) investigate the relationship between the source-sink relationship and grain yield and quality under various water strategies, and (3) recommend an appropriate irrigation strategy.

## Materials and methods

### Experimental site description and rice growth conditions

The study was conducted in 2021 at the Tianye Agricultural Research Institute in Shihezi, Xinjiang Uygur Autonomous Region, China (86°1′12″E, 44°33′0″N, 412 meters above sea level). Total precipitation during the rice-growing season was 71.1 mm (data from a local meteorological station). The basic physical and chemical properties of the soil were as follows: field water capacity of 26.51%, soil bulk density of 1.30 g·cm^-3^, soil organic matter of 3.79%, alkali hydrolyzable N of 102.0 mg·kg^-1^, available P of 41.8 mg·kg^-1^, and available K of 176.0 mg·kg^-1^. The variety studied was Liangxiang 3 (*Oryza sativa* L.).

### Experimental design

**Figure 1:**
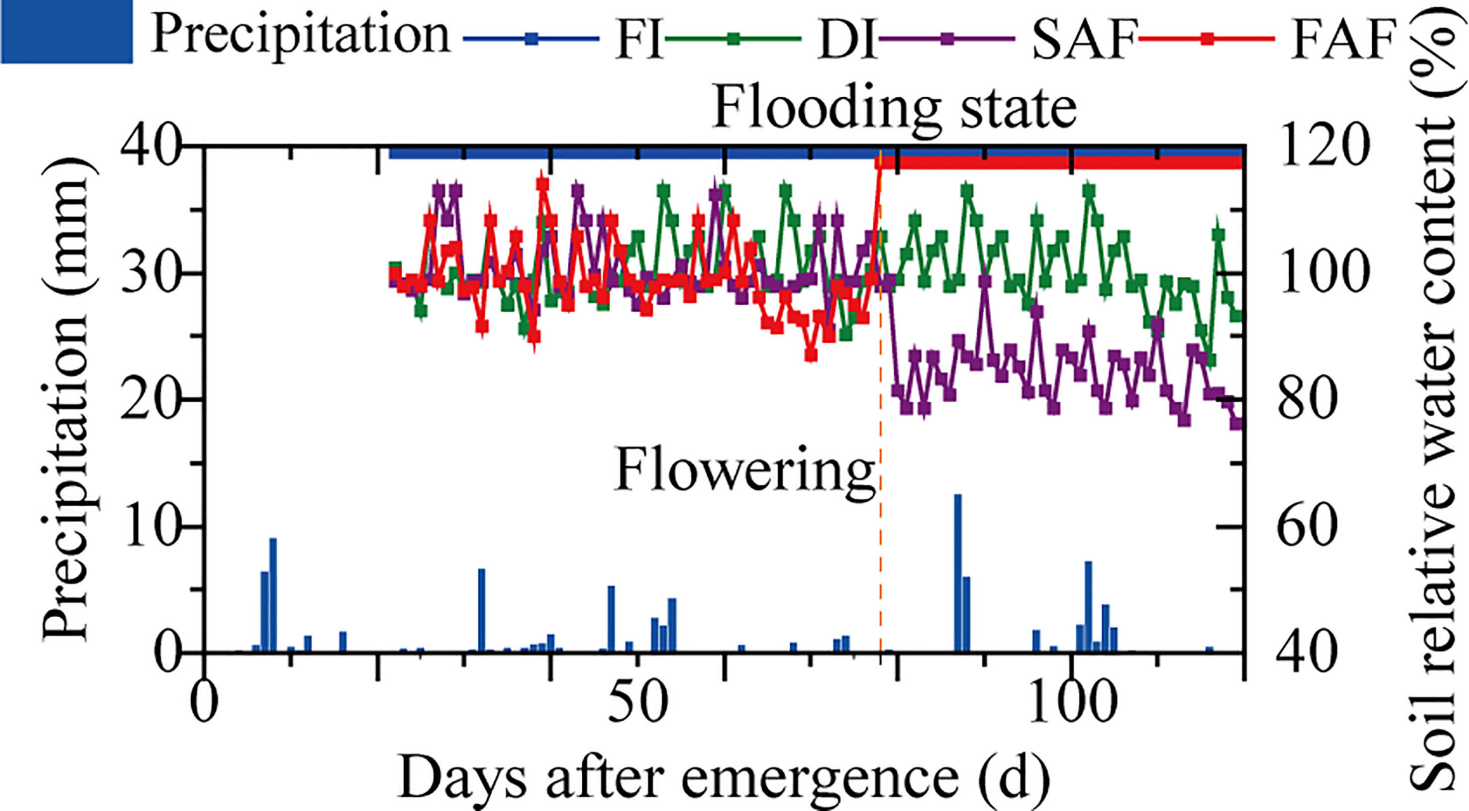
Dynamics of precipitation and soil relative water content in four water strategies during rice growing season (Xinjiang Tianye Agricultural Research Institute, Northwest China, 2021). FI, the whole growing season flooding; DI, the whole growing season normal drip irrigation (soil relative moisture (RSM) was maintained in a range of 90–100%); SAF, pre-anthesis normal drip irrigation and post-anthesis water stress (the RSM was maintained in a range of 80–90% after anthesis); FAF, pre-anthesis normal drip irrigation and post-anthesis flooding.

This experiment adopted a randomized block design with three replicates and each plot area was 30 m^2^ (each plot was 12.5 m long and 2.4 m wide, including eight rows). The four treatments were as follows: (1) FI, flooding with a 3-6 cm water layer during the whole rice growth season; (2) DI, normal drip irrigation (the relative soil moisture (RSM) was maintained in a range of 90-100%); (3) SAF, normal drip irrigation before anthesis, water stress after anthesis (the RSM was maintained at a range of 80-90%); and (4) FAF, normal drip irrigation before flowering and flooding after flowering. The fertilizer was drip-irrigated into the plots. A drip irrigation system was installed in the FI and FAF plots for fertilization, and an irrigation pipe (opened after fertilization) was installed. There was a short period of water drying before fertilization (the soil was still saturated). The plots of the four irrigation strategies received identical amounts of fertilizer and the same fertilization patterns were used (N, 300; P_2_O_5_, 110; K_2_O, 70 kg·hm^-2^). A water meter was connected to the valve of each treatment pipeline to record the irrigation volume. During the whole growth season, the irrigation volumes of the FI, DI, SAF, and FAF treatments were 18300.0, 10558.6, 9470.3, and 13503.2 m^3^·hm^-2^, respectively. TDR (time-domain reflectometer, TRIME-TDR, IMKO, Germany) was used to monitor the soil moisture content. During the growing season, the soil water content was monitored at a fixed time each day (09:00 am). Supplemental irrigation was initiated when the soil water content was below the lowest irrigation threshold for the corresponding treatment. The highest irrigation threshold for the treatment was achieved when the soil water content reached this level. **Figure 1** depicts the dynamic soil water content and local precipitation under various water strategies during the rice-growing season.

The seeds were sown on April 24, 2021 at 2-3 cm depth, with plant spacing of 10 cm, row spacing of 0.26 m, six to eight seeds per hill, and a density of 3.0 × 10^5^ hill·hm^-2^. One drip pipe serviced two rows of rice drip irrigation. Before sowing, pools were dug with a depth of 0.6 m and an area of 20 m^2^, and then the soil was backfilled layer-by-layer after embedding the impervious film as plots for FI and FAF treatments.

### Investigating the source-sink relationship during the grain filling stage

When 50% of the panicles appeared in the flag leaf sheath, 50 hills of rice plants were randomly selected from each plot, and three tillers were labeled with a signboard in each hill. Shoots of six labeled tillers were sampled every 7 d from flowering to maturity, shoots of six labeled tillers were sampled and divided into vegetative parts and panicles. These samples were first heated at 105 °C, then were dried at 75 °C, and the dry weight of these two parts were weighed. Then the number of grains, the weight per grain (grains weight/number of grains per panicle) and the weight of above-ground plants per grain were calculated (number of grains per panicle/vegetative parts weight) (Shi et al., 2017; Pan et al., 2022).

According to Yin (2002), the following sigmoid growth function was used to simulate temporal changes in grain weight (W_grain_) over time (t) after flowering:


(1) $$\begin{array}{c} W_{grain}=\{\begin{array}{c} W_{b}+\left(W_{max}-W_{b}\right)\left(1+\frac{t_{e}-t}{t_{e}-t_{m}}\right){\left(\frac{t-t_{b}}{t_{e}-t_{b}}\right)}^{\frac{\left(t_{e}-t_{b}\right)}{\left(t_{e}-t_{m}\right)}},\;t_{b}\leq t\leq t_{e}\\ W_{max},\;t>t_{e} \end{array} \end{array}$$

where, t is the day after 100% flowering, W_b_ is the initial grain weight at the beginning of grain filling, t_b_ is the time when grains start to fill, t_e_ is the time when grain filling ends, W_max_ is the maximum weight of the grains, t_m_ is the time taken to reach the maximum filling rate (G_m_). The maximum grain filling rate G_m_ was calculated as follows (Shi et al., 2016):


(2) $$\begin{array}{c} G_{m}=\frac{2t_{e}-t_{m}}{t_{e}\left(t_{e}-t_{m}\right)}{\left(\frac{t_{m}-t_{b}}{t_{e}-t_{b}}\right)}^{\frac{t_{m}-t_{b}}{t_{e}-t_{m}}}\left(W_{max}-W_{b}\right) \end{array}$$

Pan et al. (2022) suggested that the sink activity and sink growth during the grain-filling stage can be calculated as follows:


(3) $$Sink\;activity=G_{m}\left(\frac{t_{e}-t}{t_{e}-t_{m}}\right){\left(\frac{t}{t_{m}}\right)}^{\frac{t_{m}}{t_{e}-t_{m}}}$$

(4) $$Sink\;growth=W_{max}-W_{b}$$

According to Yin et al. (2009), temporal changes in the whole above-ground plant weight (W_plant_) after 100% flowering were simulated using the following sigmoid growth function:


(5) $$W_{plant}=\{\begin{array}{cc} S_{max}t\left\{1-\left(\frac{1}{2t_{e0}-t_{m0}}\right){\left(\frac{t}{t_{e0}}\right)}^{\frac{t_{e0}}{t_{e0}-t_{m0}}}\left[\left(2t_{e0}-t_{m0}-t\right)+\frac{\left(t_{e0}-t_{m0}\right)\text{t}}{3t_{e0}-2t_{m0}}\right]\right\}+W_{h}, & t<t_{e0}\\ W_{h}+S_{max}\frac{t_{e0}{}^{2}}{3t_{e0}-2t_{m0}}, & t\geq t_{e0} \end{array}$$

where t is the number of days after 100% flowering, W_h_ is the initial weight of the above-ground plants at 100% flowering, t_m0_ is the time when the source activity decreases the fastest, t_e0_ is the time when the source activity is zero, and *S_max_* is the maximum source activity. W_h_, t_m0_, t_e0_ and S_max_ were estimated by fitting Eqs. (5) to the measured data for the temporal changes in W_plant_.

The source activity and capacity were simulated according to the function described by Yin et al. (2009):


(6) $$\begin{array}{c} Source\;activity=\{\begin{array}{c} S_{max}\left[1-\left(1+\frac{t_{e0}-t}{t_{e0}-t_{m0}}\right){\left(\frac{t}{t_{e0}}\right)}^{\frac{t_{e0}}{\left(t_{e0}-t_{m0}\right)}}\right],\;t<t_{e0}\\ 0,\;t\geq t_{e0} \end{array} \end{array}$$

(7) $$Source\;capacity=S_{max}\frac{t_{e0}{}^{2}}{3t_{e}-t_{m}}$$

According to Pan et al. (2022), the obtained sink-source difference was obtained by subtracting the source capacity from the sink growth, and the source/sink ratio was obtained from the source capacity/sink growth (based on the same calculation principle and unit).

### Yield, yield components, harvest index and water productivity

The grain yield at the maturity stage was estimated from three 1 m^2^ areas in each plot (excluding the marginal effect). Fifteen hills from each plot were used to determine the yield composition, including the percentage of productive tillers (productive tillers are tillers that bear fertile panicles), spiklets per panicle, seed-setting rate, 1000 grain weight and above-ground biomass. According to Ghasemi-Aghbolaghi and Sepaskhah (2017), water productivity (WP) was obtained by dividing the grain yield by the irrigation amount. The harvest index (HI) was obtained by dividing the grain yield by the above-ground biomass.

### Determination of key enzymes for starch synthesis in grains on different panicle positions

**Figure 2:**
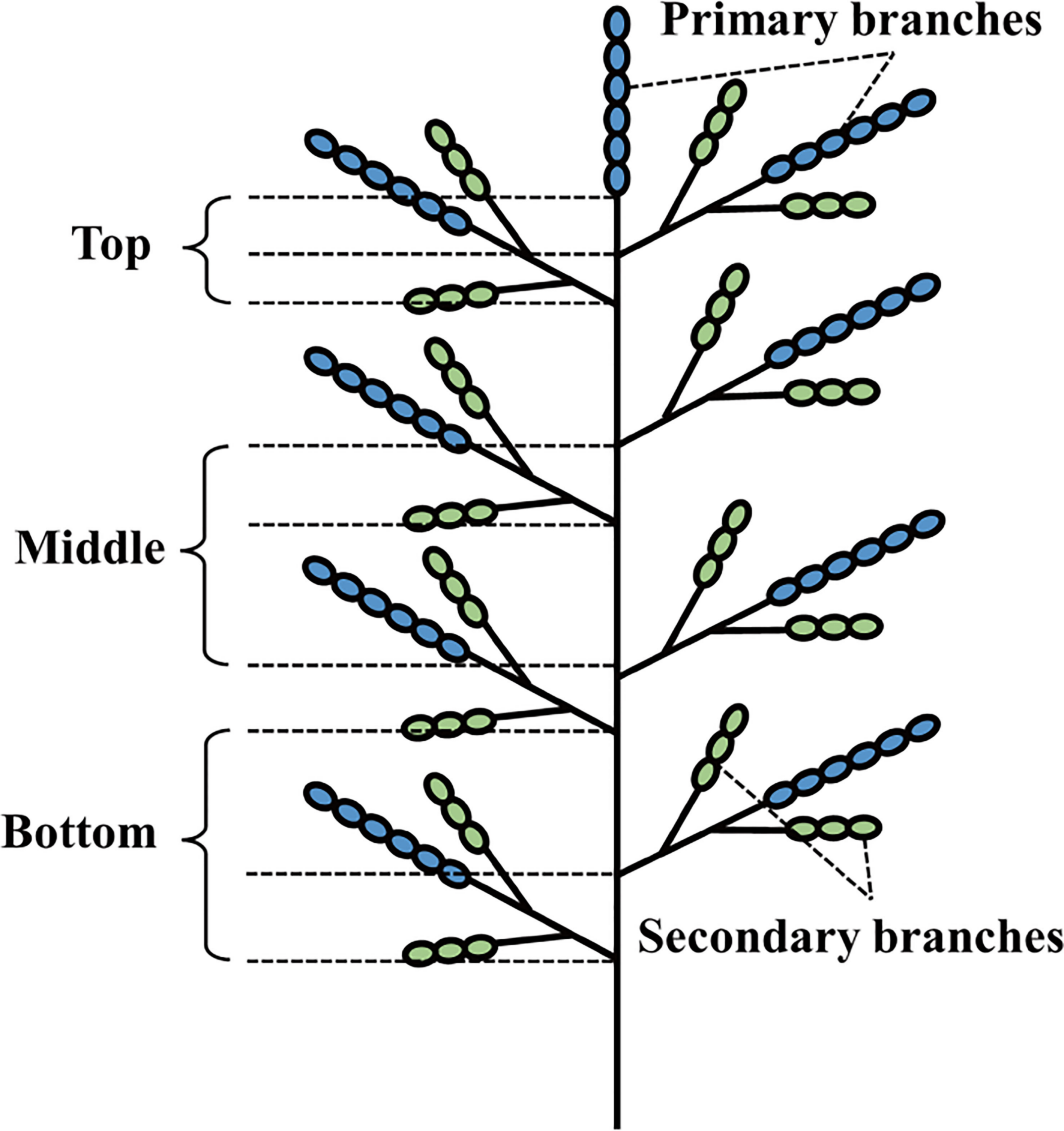
Structure schematic of a panicle.

Twenty panicles that flowered on the same day were labeled in each plot. At the middle filling stage, 10 labeled panicles were taken and immediately placed in liquid nitrogen for 1 min, and then stored at -80 °C to determine the activities of ADP-glucose pyrophosphorylase (AGPase), granular starch synthase (GBSS), starch branching enzyme (SBE), and starch debranching enzyme (DBE). The panicle division method described by You et al. (2021) was modified slightly, and the rice panicle was divided into six parts: primary and secondary branches at the top of the panicles, primary and secondary branches in the middle of the panicles, and primary and secondary branches at the bottom of the panicles (**Figure 2**). The sample was taken from the ultra-low temperature refrigerator, 0.1–0.2 g of the sample was weighed, 1 mL of the extract was added, and the sample was then homogenized in an ice bath. The sample was then centrifuged at 10000 × *g* at 4 °C for 10 min and the supernatant was discarded, 1 mL of extract was added to the sediment, which was completely mixed, and then put on ice for testing. Kit boxes were used to measure enzyme activity (Shanghai Enzymelinked Biotechnology Co., Ltd.).

### Amylose, amylopectin, total protein and protein components in grains on different panicle positions

Fifty panicles flowering on the same day were labeled in each plot, removed at maturity, and divided into six parts according to the method described above. The grains of the six parts were removed and peeled, and a ball mill was used to grind the grains into rice flour for testing. A kit box was used to determine the amylose and amylopectin contents of the samples. The method details are as follows: 0.01–0.02 g of dried sample was weighed (approximately 0.01 g is recommended) and ground in a mortar, 1 mL of reagent I was added, then the sample was fully homogenized and transferred to an EP tube. The sample was then extracted in a water bath at 80 °C for 30 min, 3000 × *g*, centrifuged at 25 °C for 5 min, and the supernatant was discarded, and the precipitant was retained. Then, 1 mL of reagent II (ether) was added, the sample was shaken for 5 min, then centrifuged at 3000 × *g*, 25 °C for 5 min, and the supernatant was discarded, and the precipitant was retained. After, 1 mL of reagent III was added and the precipitant was fully dissolved, then heated in a water bath at 90 °C for 10 min and cooled for testing (the method descriptions for the two samples were the same, but the reagents were different).

The Kjeldahl method was then used to determine the total N content of the above samples, which were converted into total protein content (conversion coefficient: 5.95). The content of protein components was determined (albumin, globulin, gliadin, and glutenin) according to the method described by Takemoto et al. (2002).

### Statistical analysis

All data were processed using Excel 2019. Analysis of variance was performed using SPSS software (version 20.0; SPSS, Chicago, IL, USA). The statistical models included irrigation strategies (I), panicle positions (P), and their interactions (I × P). Means were tested using the least significant difference test at P< 0.05 (LSD0.05). The figures and model fitting (Eq. (1) and Eq. (5)) were performed using OriginPro 22.0 software (OriginLab Corporation, Northampton, MA, USA).

## Result

### Yield, yield components, harvest index and water productivity

Post-anthesis, different irrigation strategies significantly affected rice yield, water productivity, and harvest index (**Table 1**). The FI yield was significantly greater than that of DI and SAF; however, there was no significant difference between FI and FAF. Compared to DI, SAF significantly reduced the yield by 15%, and FAF increased it by 29.2%. Compared with the FI treatment, DI, SAF, and FAF saved 42.3%, 48.2%, and 26.2% of irrigation water, respectively. FI and FAF significantly reduced the WP compared to DI and SAF. The WP of FAF was significantly increased by 19% compared with that of FI. The highest HI was obtained by FAF, and no significant difference was observed between FAF and FI. The HI of SAF was significantly reduced by 12.5% and that of FAF was significantly increased by 11% compared with DI. The above results show that post-anthesis further limiting irrigation significantly reduced the yield and HI of drip-irrigated rice, whereas post-anthesis supplementary irrigation increased the yield, HI and WP of drip-irrigated rice.

**Table 1:** Yield, harvest index (HI) and water productivity (WP) in different irrigation strategy treatments.

Irrigation strategies	Yield (t·hm^-2^)	Water productivity (kg·m^-3^)	Harvest index
FI	9.94 ± 0.56 a	0.55 ± 0.03 c	0.43 ± 0.01 a
DI	7.70 ± 0.08 b	0.73 ± 0.07 a	0.40 ± 0.01 b
SAF	6.54 ± 0.08 c	0.71 ± 0.02 a	0.35 ± 0.01 c
FAF	9.24 ± 0.58 a	0.68 ± 0.01 b	0.45 ± 0.02 a

The data are mean ± standard errors of three replicates. Different letters indicate statistical significance at the P = 0.05 level within the same column, value are means (n = 3). The Least-Significant Difference Test (LSD) was used for comparison. FI, the whole growing season flooding; DI, the whole growing season normal drip irrigation (soil relative moisture (RSM) was maintained in a range of 90–100%); SAF, pre-anthesis normal drip irrigation and post-anthesis water stress (the RSM was maintained in a range of 80–90% after anthesis); FAF, pre-anthesis normal drip irrigation and post-anthesis flooding

We further analyzed the yield components of rice under the four irrigation strategies (**Table 2**). The percentage of productive tillers in the FI treatment was significantly greater than that in the DI, SAF, and FAF treatments, indicating that drip irrigation before anthesis increased the number of unproductive tillers. The percentage of productive tillers in SAF was significantly reduced by 7.9% compared with DI, whereas there was no significant difference between DI and FAF, indicating that post-anthesis further restricted irrigation aggravated the reduction in tillering of drip-irrigated rice, whereas post-anthesis supplementary irrigation had no effect on the percentage of productive tillers.

**Table 2:** Yield components in different irrigation strategy treatments.

Irrigation strategies	Percentage of productive tiller (%)	Spikelets per panicle	Seed-setting rate (%)	1000-grain weight (g)
FI	91.8 ± 0.9 a	94.3 ± 2.1 a	82.5 ± 1.0 a	31.2 ± 0.3 a
DI	83.1 ± 3.0 b	86.6 ± 2.2 b	74.8 ± 1.2 b	28.3 ± 1.4 b
SAF	76.5 ± 2.8 c	84.8 ± 3.7 b	72.4 ± 1.3 c	26.0 ± 0.4 c
FAF	85.3 ± 2.1 b	85.2 ± 2.1 b	83.0 ± 0.6 a	29.9 ± 0.6 a

The data are mean ± standard errors of three replicates. Different letters indicate statistical significance at the P = 0.05 level within the same column, value are means (n = 3). The Least-Significant Difference Test (LSD) was used for comparison. FI, the whole growing season flooding; DI, the whole growing season normal drip irrigation (soil relative moisture (RSM) was maintained in a range of 90–100%); SAF, pre-anthesis normal drip irrigation and post-anthesis water stress (the RSM was maintained in a range of 80–90% after anthesis); FAF, pre-anthesis normal drip irrigation and post-anthesis flooding.

Spikelets per panicle in DI, SAF, and FAF decreased significantly compared to those in FI, but there was no significant difference among the three treatments, indicating that the number of spikelets per panicle was determined before anthesis. The seed-setting rate and 1000 grain weight of the four treatments showed similar trends (**Table 2**). The seed-setting rate and 1000 grain weight of the SAF treatment decreased by 3.2% and 8.0%, respectively, and those of the FAF treatment increased by 12.8% and 13.0%, respectively, compared to DI. No significant differences were observed between the FI and FAF groups. These results indicate that post-anthesis further restricted irrigation seriously reduced the seed-setting rate and 1000 grain weight of drip-irrigated rice, whereas post-anthesis supplementary irrigation improved the seed-setting rate and 1000 grain weight.

**Figure 3:**
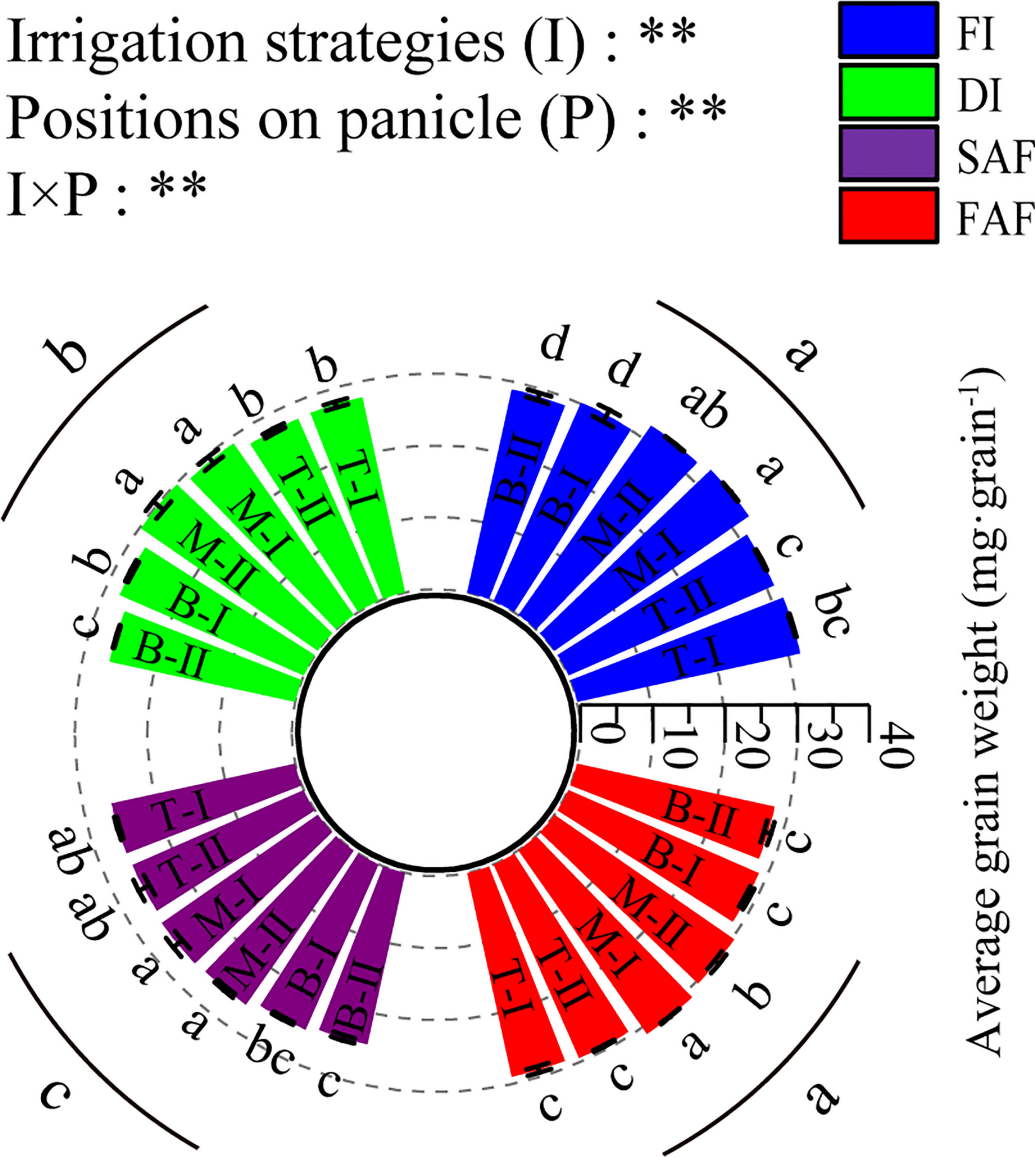
Average grain weight on different positions of panicles. The vertical line on the column represents three repeated standard errors. Different letters represent significant difference among treatments (P< 0.05). The Least-Significant Difference Test (LSD) was used for comparison. ANOVA of treatment (T) and positions of panicle (P) and their interaction (T × P) (**P<0.01). FI, the whole growing season flooding; DI, the whole growing season normal drip irrigation (soil relative moisture (RSM) was maintained in a range of 90–100%); SAF, pre-anthesis normal drip irrigation and post-anthesis water stress (the RSM was maintained in a range of 80–90% after anthesis); FAF, pre-anthesis normal drip irrigation and post-anthesis flooding; T, M and B represent the top, middle and bottom of the panicles respectively, and the Roman numerals I and II represent the primary and secondary branches respectively.

As shown in **Figure 3**, the average weight of the grains in the middle of the panicles in the four treatments was significantly greater than that at the top and bottom of the panicles. The grain weight of the secondary branches at the bottom of the panicles significantly decreased by 11.5% compared to that in the middle of the panicles. The average grain weights of the bottom secondary branches of the rice panicles in FI and FAF were significantly higher than those in DI and SAF, and those of the bottom secondary branches of the rice panicles in SAF were the lowest. Compared with DI, SAF reduced the average grain weight of the secondary branches at the bottom of panicles by 7%, and FAF increased the average grain weight in the lower part of the panicle by 2.6%, which indicates that post-anthesis water stress has the greatest impact on the grain filling quality at the bottom of panicles.

### Source-sink relationship during the grain filling period

**Figure 4:**
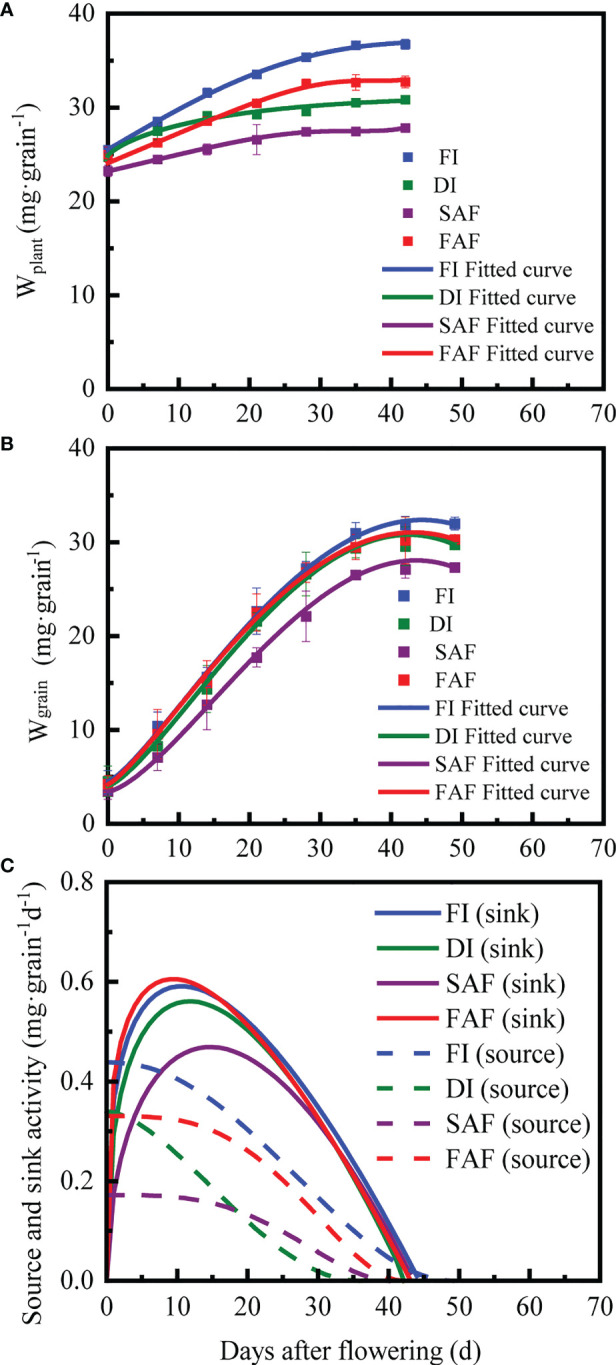
The observed mean value (W_grain_ and W_plant_) and fitting curve of grain weight **(A)** and vegetable parts of shoots weight per grain **(B)** during grain filling stage in different irrigation strategies; dynamics of source-sink activity during grain filling stage (solid line indicates sink activity, dotted line indicates source activity) **(C)**. FI, the whole growing season flooding; DI, the whole growing season normal drip irrigation (soil relative moisture (RSM) was maintained in a range of 90–100%); SAF, pre-anthesis normal drip irrigation and post-anthesis water stress (the RSM was maintained in a range of 80–90% after anthesis); FAF, pre-anthesis normal drip irrigation and post-anthesis flooding.

According to the R^2^ range of the source parameters (0.887-0.999) in **Table 3**, Formula (5) well-fitted the dynamic change in the above-ground plant weight (W_plant_) during the grain-filling period (**Figure 4A**). Compared with FI, DI and FAF, the initial above-ground plant weight (W_h_) of SAF decreased by 3.1-8.9% (P< 0.05) and maximum source activity (S_max_) of SAF significantly decreased by 47.9-60.7% (P< 0.05). Compared to FI, SAF, and FAF, the time of source activity (t_e0_) of DI was reduced by 5-15 days, and the time of rapid reduction of source activity (t_m0_) increased by approximately 15 days. In general, there were no significant differences in the source parameters between FI and FAF, and the SAF parameters were the lowest among all treatments.

**Table 3:** Estimated parameters of rice sink activity and source activity in different irrigation strategy treatments.

Irrigation strategies	Parameters of sink							Parameters of source				
	W_max_	W_b_	t_m_	t_e_	G_m_	G_ave_	R^2^	S_max_	W_h_	t_m0_	t_e0_	R^2^
FI	32.4 ± 0.1 a	4.52 ± 0.49 a	10.6 ± 1.7 bc	44.3 ± 0.6 a	0.927 ± 0.037 a	0.568 ± 0.012 a	0.998	0.438 ± 0.004 a	25.5 ± 0.1 a	27.4 ± 0.5 ab	49.0 ± 1.03 a	0.999
DI	30.8 ± 0.5 b	4.04 ± 0.83 ab	11.8 ± 0.8 ab	42.5 ± 0.8 a	0.917 ± 0.055 a	0.546 ± 0.023 b	0.997	0.340 ± 0.041 ab	24.8 ± 0.1 a	14.8 ± 4.1 c	35.8 ± 0.80 b	0.887
SAF	28.1 ± 0.2 c	3.44 ± 0.29 b	14.7 ± 1.0 a	43.6 ± 2.5 a	0.816 ± 0.036 b	0.502 ± 0.001 c	0.998	0.172 ± 0.050 c	23.3 ± 0.5 b	29.0 ± 4.1 b	40.3 ± 0.61 ab	0.983
FAF	31.0 ± 0.4 b	4.22 ± 0.2 ab	8.49 ± 2.4 c	43.1 ± 0.6 a	0.927 ± 0.055 a	0.548 ± 0.005 ab	0.999	0.330 ± 0.006 b	24.0 ± 0.6 a	30.1 ± 0.7 a	42.2 ± 3.38 a	0.990

The data are mean ± standard errors of three replicates. Different letters indicate statistical significance at the P = 0.05 level within the same column, value are means (n = 3). The Least-Significant Difference Test (LSD) was used for comparison. FI, the whole growing season flooding; DI, the whole growing season normal drip irrigation (soil relative moisture (RSM) was maintained in a range of 90–100%); SAF, pre-anthesis normal drip irrigation and post-anthesis water stress (the RSM was maintained in a range of 80–90% after anthesis); FAF, pre-anthesis normal drip irrigation and post-anthesis flooding; W_max_ represents the grain maximum grain weight (mg·grain^-1^); W_b_ represents the initial weight of grain; t_m_ represents the days (d) to reach the maximum sink activity; t_e_ represents the days (d) when the sink activity is zero; G_m_ represents the maximum grain filling rate (mg·grain^-1^·d^-1^); G_ave_ represents the average grain filling rate (mg·grain^-1^·d^-1^); S_max_ represents the maximum source activity (mg·grain^-1^·d^-1^); W_h_ represents the initial value of the shoots biomass per grains (mg·grain^-1^); t_m0_ indicates the days (d) when the source activity decreases fastest; t_e0_ represents the days when the source stops supplying (d).

According to the *R^2^* range of sink parameters (0.997-0.999) in **Table 3**, Formula (1) well-fitted the dynamic change in grain weight with time (**Figure 4B**). The time at which the grains began to fill (t_b_) was set to zero. Compared with FI, DI, and FAF, the maximum grain weight (W_max_) of SAF decreased by 13.3%, 8.9%, and 9.6%, respectively. The time to reach W_max_ (t_e_) was not significantly different between the four treatments, but the time taken to reach the maximum grain filling rate (t_m_) in SAF was longer by approximately 5 days than the other treatments. In general, SAF resulted in a significantly reduced maximum grain filling rate (G_m_) and average grain filling rate (G_ave_). The grain filling rates of DI and SAF were inferior to that of FI and FAF.

### The dynamics of sink and source activity

The dynamics of source and sink activities are shown in **Figure 4C**. Sink growth represents the total amount of assimilates accumulated during the grain-filling stage, source capacity represents the sum of assimilates produced by vegetative organs after anthesis; assimilates stored by vegetative organs before anthesis are transferred to grains (**Table 4**). The sink growth of the SAF samples significantly decreased by 7.9–11.6% compared with that of the FI, DI, and FAF treatments (**Table 4**). The sink growth of the FAF and DI samples was significantly lower than that of FI; however, this decline was not as dramatic as that of SAF.

**Table 4:** Sink growth, source capacity and source-sink balance in different irrigation strategies.

Irrigation strategies	Sink growth (mg·grain^-1^)	Source capacity (mg·grain^-1^)	Sink-source difference (mg·grain^-1^)	Source/sink ratio
FI	27.85 ± 0.98 a	11.41 ± 0.57 a	16.44 ± 0.41 d	0.41 ± 0.03 a
DI	26.74 ± 0.26 b	5.60 ± 1.11 c	21.14 ± 0.79 a	0.21 ± 0.01 c
SAF	24.62 ± 0.4 c	4.44 ± 0.07 c	20.17 ± 0.47 b	0.18 ± 0.01 c
FAF	26.83 ± 0.34 b	8.87 ± 0.24 b	17.96 ± 0.11 c	0.33 ± 0.01 b

The data are mean ± standard errors of three replicates. Different letters indicate statistical significance at the P = 0.05 level within the same column, value are means (n = 3). The Least-Significant Difference Test (LSD) was used for comparison. FI, the whole growing season flooding; DI, the whole growing season normal drip irrigation (soil relative moisture (RSM) was maintained in a range of 90–100%); SAF, pre-anthesis normal drip irrigation and post-anthesis water stress (the RSM was maintained in a range of 80–90% after anthesis); FAF, pre-anthesis normal drip irrigation and post-anthesis flooding. Sink-source difference = Sink growth - Source capacity; Source/sink ratio = Source capacity/Sink growth

At the early stage of grain filling, source activity was greater than sink activity in all treatments (**Figure 4C**). In most cases, the sink activity in all treatments was greater than the source activity until the middle and later grain-filling stages, indicating that assimilates stored in vegetative organs began to be transported to the grains here. Compared with DI, the source capacity of SAF and FAF decreased by 20.7% and increased by 36.9%, respectively (**Table 4**). The source activity of FI was the highest among all the treatments (**Figure 4C**). In addition, FAF prolonged the source activity time (2-7 days) compared to DI and SAF, and the source activity of DI decreased the fastest. Although the source activity time of SAF was longer than that of DI, its initial value was lower (**Figure 4C**).

The source-sink balance can be described by the sink-source difference (sink growth minus source capacity, mg·grain^-1^) or the source/sink ratio (source capacity/sink growth). As shown in **Table 4**, the source-sink balance was significantly affected by the different irrigation strategies. The positive difference between the sink and source (**Table 4**) indicated that the source was the limiting factor for the grain yield of irrigated rice. DI, SAF, and FAF significantly increased the sink-source difference compared with FI (**Table 4**). FAF showed a significant decrease in the sink-source difference compared with DI and SAF. Among all treatments, the sink-source difference in FI was the smallest, and the source/sink ratios in DI and SAF were the smallest. The S/S ratio of FI was the largest among the four treatments, and the S/S ratio of FAF was 36.4% higher than that of DI.

### Key enzymes for starch synthesis in grains

**Figure 5:**
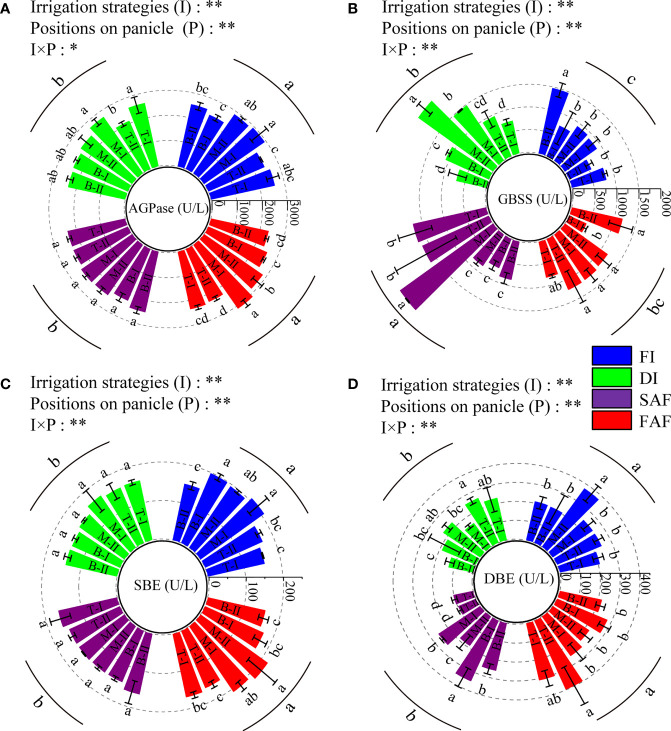
ADP-glucose pyrophosphorylase activity **(A)**, granular starch synthase activity **(B)**, starch debranching enzyme activity **(C)** and starch branching enzyme activity **(D)** in grains on different positions of panicles at the middle grain filling stage. The catalytic production of 1μmol NADPH per minute by 1L reaction system is defined as one enzyme unit (U/L). The vertical line on the column represents three repeated standard errors. Different letters represent significant difference among treatments (P< 0.05). The Least-Significant Difference Test (LSD) was used for comparison. ANOVA of treatment (T) and positions of panicle (P) and their interaction (T × P) (**P< 0.01; *P< 0.05). FI, the whole growing season flooding; DI, the whole growing season normal drip irrigation (soil relative moisture (RSM) was maintained in a range of 90–100%); SAF, pre-anthesis normal drip irrigation and post-anthesis water stress (the RSM was maintained in a range of 80–90% after anthesis); FAF, pre-anthesis normal drip irrigation and post-anthesis flooding; T, M and B represent the top, middle and bottom of the panicles respectively, and the Roman numerals I and II represent the primary and secondary branches respectively.

AGPase limits starch synthesis. As shown in **Figure 5A**, the AGPase activity in grains of FI and FAF was significantly greater than that of DI and SAF. The AGPase activity of the grains in the middle of the panicles in FI and FAF was greater than that in the top and bottom of the panicles. However, the AGPase activity in DI and SAF grains was not significantly different among grains at different panicle positions.

The GBSS activity is closely related to amylose synthesis. As shown in **Figure 5B**, the GBSS activity of SAF was significantly higher than that of FI, DI, and FAF, and the GBSS activity of FI was the lowest. The GBSS activity of grains on the top and middle primary branches of panicles of SAF was significantly greater than that of grains in other positions, and that of grains in the middle of panicles of DI was significantly greater than that of grains in other positions. The GBSS activity of the grains at different panicle positions showed little difference between FI and FAF.

The activities of SBE and DBE are closely related to amylopectin synthesis. As shown in **Figures 5C, D**, compared with DI and SAF, SBE activity in the grains of FI and FAF was significantly increased. The SBE activity of grains in the middle of panicles in FI and FAF was greater than that of grains in other positions of the panicles and also greater than that of grains in the same or different positions in the panicles of other treatments. The activity of DBE in the grains at different positions in the rice panicle differed, but there was no obvious pattern. In general, the SBE and DBE activities in the grains of the FI and FAF treatments were significantly greater than those of the DI and SAF treatments.

In conclusion, the activity of AGPase, SBE, and DBE in rice grains of SAF showed no significant difference compared to DI, whereas those of FAF were significantly increased, and there was no significant difference between FI and FAF. The activities of the four starch synthetases in grains at different panicle positions were significantly different, whereas the activities of the starch synthetases in grains in the middle panicles of rice were generally greater.

### Amylose, amylopectin, crude protein and protein components in grains

**Figure 6:**
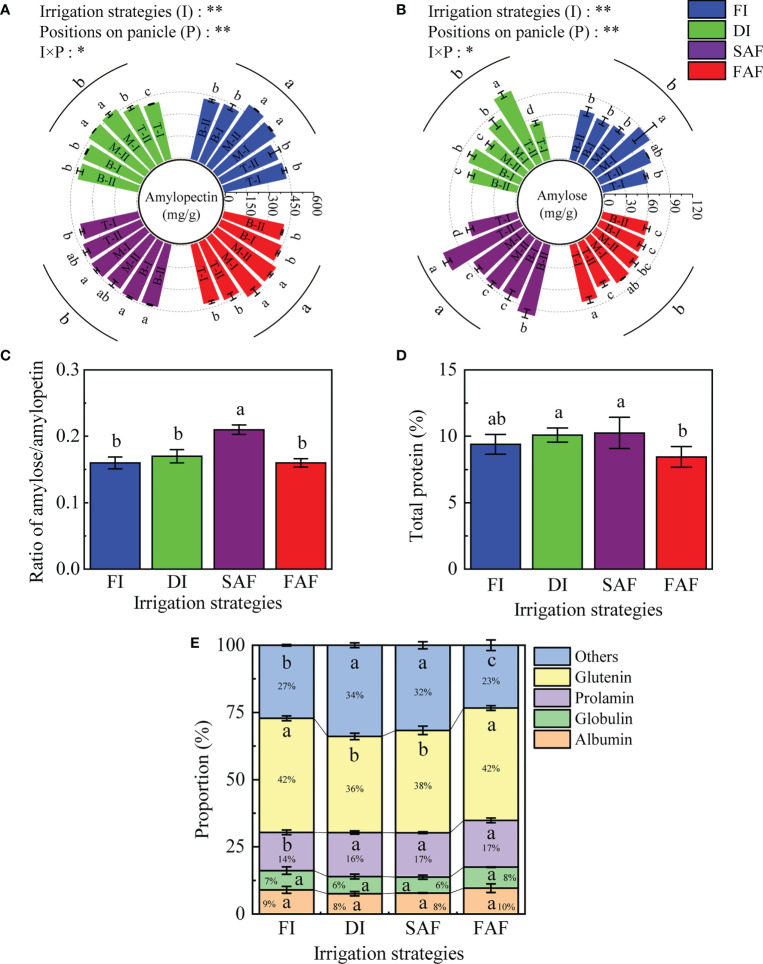
Amylopectin content **(A)** and amylose content **(B)** in grains on different positions of panicles in four water strategy management; The ratio of amylose content/amylopectin content **(C)**; Total protein content in grains **(D)** and proportion of protein components to total protein content **(E)**. The vertical line on the column represents three repeated standard errors. Different letters represent significant difference among treatments (P< 0.05). The Least-Significant Difference Test (LSD) was used for comparison. ANOVA of treatment (T) and positions of panicle (P) and their interaction (T × P) (**P< 0.01; *P< 0.05). FI, the whole growing season flooding; DI, the whole growing season normal drip irrigation (soil relative moisture (RSM) was maintained in a range of 90–100%); SAF, pre-anthesis normal drip irrigation and post-anthesis water stress (the RSM was maintained in a range of 80–90% after anthesis); FAF, pre-anthesis normal drip irrigation and post-anthesis flooding; T, M and B represent the top, middle and bottom of the panicles respectively, and the Roman numerals I and II represent the primary and secondary branches respectively.

As shown in **Figure 6A**, the amylopectin content in the grains of FI and FAF was significantly greater than that in DI and SAF. Compared to the other three treatments, SAF increased the amylose/amylopectin ratio in grains by approximately 23.8% (**Figure 6C**). The amylopectin content in grains in the middle of the panicles was significantly higher than those in other positions of the panicles in FI and FAF, which was consistent with the trend of SBE activity (**Figure 5C**). As shown in **Figure 6B**, the amylose content of SAF was significantly higher than that of DI, FAF, and FI, and the amylose content of the grains on the top and bottom secondary branches of the panicles increased significantly. The amylose content in grains of FAF and FI was similar and slightly lower than that of DI, which indicates that post-anthesis further water restriction increased the amylose content in rice grains under drip irrigation management.

The data in this study showed that the changes in protein content and protein components in grains with different panicle positions followed no obvious trends under the different irrigation strategies (**Supplementary Dataset Table S1**). Overall, the total protein content of grains of the SAF treatment was slightly higher than that in the FI and DI treatments; however, the difference was not significant (**Figure 6D**). Compared with DI and SAF, the total protein content of the grains in FAF decreased by 16.3% (P< 0.05) and 17.6% (P< 0.05), respectively. The gliadin content in the grains of FI was significantly lower than that in DI and SAF, and the difference in gliadin content between FI and FAF was not significant. The albumin and globulin contents of DI and SAF were lower than those of FI and FAF, and there was no difference in gluten content among the four treatments. As shown in **Figure 6E**, the proportion of gliadin in the total protein of DI, SAF, and FAF was greater than that of the FI. The proportion of glutenin in the total protein of SAF and DI was 9.5–14.3% lower than that of FI and FAF. SAF and DI also reduced the proportion of albumin and globulin in total protein.

## Discussion

### Source-sink relationships in different water strategy managements

Only a few studies have reported rice source-sink changes under different water conditions during the filling period. The reduction in source intensity during drought stress affects the source-sink relationship of plants, leading to a decrease in yield (Peleg et al., 2011). This study found that post-anthesis irrigation strategies had a significant impact on sink growth. Compared with DI, the sink growth of SAF was significantly reduced and the source capacity of FAF was significantly increased (**Table 3**, **Figure 4C**). These results may be related to the photosynthetic performance of flag leaves under growth stress. Peleg et al. (2011) pointed out that inhibiting premature senescence of leaves and increasing photosynthetic intensity can improve the source-sink intensity of plants. Our research also showed that a post-anthesis sufficient water supply improved the photosynthetic rate of flag leaves, and thus improved the source-sink intensity (**Supplementary Dataset Table S2**). In this experimental field, it is often found that, compared with the leaves of flooded rice, the leaves of drip-irrigated rice were more prone to premature senescence at the late filling stage. Previous studies have shown that a good source–sink relationship can decelerate leaf senescence and prolong the time of source activity, which balances the source-sink relationship (Kim et al., 2011; Kitonyo et al., 2018). We found that under the same nutrient conditions, flooding during the grain-filling period prolonged the duration of source activity (**Table 3**), which was conducive to the continuity of sink activity, which is consistent with the findings of Wei et al. (2018) and Pan et al. (2022). Post-anthesis, a sufficient water supply (flooding) improved the source capacity, and the sink-source difference was positive (**Table 4**), indicating that the source restriction of carbohydrates in drip-irrigated rice remained, which may also have been affected by nutrients and the climate (Wei et al., 2018; Shao et al., 2020; Pan et al., 2022).

### Source-sink balance in relation to grain yield

Previous studies have shown that the response of yield changes to environmental conditions depends on changes in source and sink traits, such as secondary branch degradation, spikelets per panicle reduction, and photosynthesis weakening (Yang et al., 2005; He et al., 2016; Moe et al., 2019). The results of this study showed that pre-anthesis drip irrigation of rice led to the degradation of tillers, whereas post-anthesis water stress (SAF) led to the degradation and death of tillers (**Table 2**), which is consistent with the findings of Sharma et al. (2012). Meanwhile, compared with DI, the seed setting rate and 1000 grain weight of FAF were significantly higher, whereas there was no difference in the percentage of productive tillers and spikelets per panicle. This demonstrates that the grain filling stage is the ideal period for maximizing rice yield and quality, confirming our hypothesis and demonstrating the validity of our experimental drip irrigation system design.

**Figure 7:**
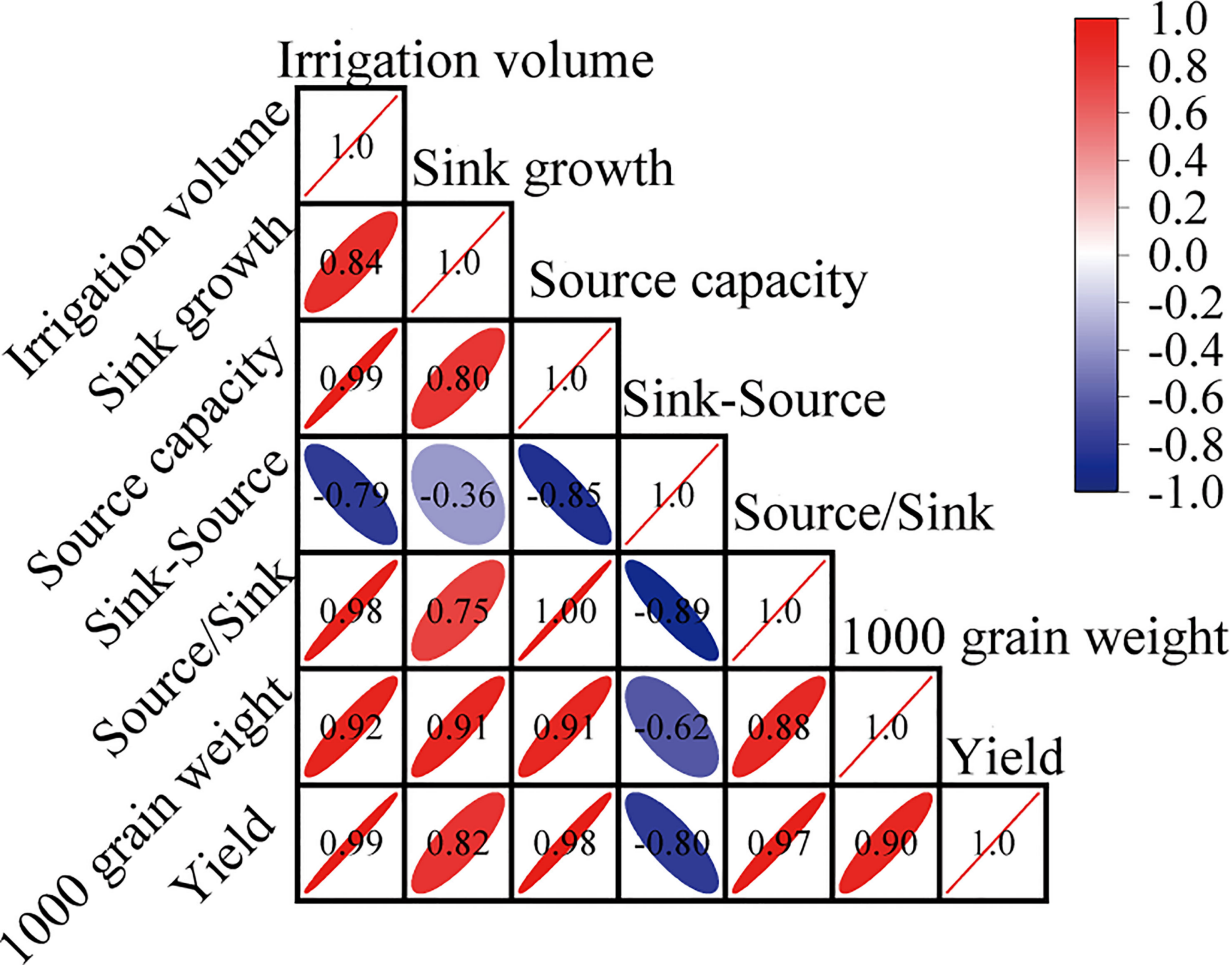
Correlation analysis among irrigation amount, source-sink balance and grain yield.

Sink-source differences (sink growth minus source capacity, mg·grain^-1^) or the source/sink ratio (source capacity/sink growth) represent transport efficiency of assimilates (Pan et al., 2022). Previous studies have shown that a balanced source-sink relationship during the grain-filling stage is crucial for crop yield. A small sink-source difference or large source/sink ratio indicates a more balanced source-sink relationship (Chang and Zhu, 2017; Wei et al., 2018). This study showed that under drip irrigation, the source-sink relationship of rice was relatively imbalanced, and post-anthesis water stress (SAF) aggravated this imbalance, leading to earlier or faster remobilization of assimilates stored in vegetative organs, ultimately leading to a rapid reduction in source activity (**Figure 4C**). Post-anthesis, different irrigation strategies had a significant impact on photosynthesis in rice flag leaves. Post-anthesis water stress significantly limited photosynthesis in the flag leaves of drip-irrigated rice, severely limiting source activity (**Supplementary Dataset Table S2**), which was consistent with the findings of Wu et al. (2018). Further analysis showed that 1000 grain weight and grain yield were negatively correlated with sink-source differences, whereas 1000 grain weight and grain yield were positively correlated with source capacity, sink growth, and the source/sink ratio (**Figure 7**). This indicated that the balancing of the source-sink relationship was conducive to the transfer of assimilates to grains to obtain a greater grain yield (**Table 1**), which was consistent with the study of Shao et al. (2020). The present study also demonstrated this point. FAF slowed the remobilization of assimilates in vegetative organs and maintained source activity by promoting a more balanced source–sink relationship (**Figure 4C**). The grain yield of SAF was severely reduced by 15% compared with that of DI, whereas the grain yield of FAF was significantly enhanced by 29.2%. (**Table 1**). In drip irrigation management, the post-anthesis maintenance of water conditions akin to flooding irrigation (FAF) was not only helpful in producing a higher grain yield and greater HI, but also improved WP by approximately 11% over normal drip irrigation. This study demonstrated that, from the perspective of irrigation to conserve water, the source-sink relationship could be modified through high-quality irrigation techniques during the grain-filling stage without considering nutrient factors, which improves the grain yield of drip-irrigated rice.

Compared with DI, the seed setting rate and 1000 grain weight of SAF were significantly reduced, whereas those of FAF were significantly increased, indicating that increasing the irrigation amount after anthesis can improve the grain-filling capacity (sink activity), thereby improving grain yield and quality. Fu et al. (2011) used two important starch synthetases (SuSase and AGPase) as indicators to measure sink activity; their results showed that the sink activity of inferior grains was easily limited, which led to a great difference between superior and inferior grain-filling capacity and ultimately restricted the grain yield. Additionally, our study confirmed that an increase in enzyme activity was conducive for improving sink activity and promoting grain filling (**Figure 3** and **Figure 5**). The data in this study showed that different irrigation strategies after anthesis had a greater impact on the grain weight at different positions of panicles; the difference in the grain weight between the middle of panicles and top/bottom of panicles was greater (**Figure 3**). The grain weight of the bottom secondary branches of panicles in SAF was reduced by 7% and that in FAF was increased by 12.9% compared with DI, indicating that the grain filling of the bottom secondary branches of panicles (inferior grains) was more sensitive to water stress (**Figure 3**). This study also found that AGPase and SBE activities in grains at different positions of the panicles were highly correlated with the average grain weight at different positions (**Supplementary Figure S1A**), which indicates that a post-anthesis sufficient water supply improved sink activity by regulating the activity of enzymes for starch synthesis in grains and ultimately promoted grain filling.

### Rice quality in different irrigation strategies

In the present study, water stress increased the total protein content of rice grains (**Figure 6D**), which is similar to the results of Song et al. (2021). This benefits the nutritional quality of rice, but high protein content has a negative impact on the taste of rice, especially the relative content of gliadin (Wang et al., 2008; Kawakatsu et al., 2010). We found that post anthesis water stress increased the proportion of gliadin and decreased the proportion of glutenin in the total protein (**Figures 6D, E**).

Amylose is not easily absorbed by the human body and negatively affects rice taste. In this study, the amylopectin and amylose contents in grains at different positions of the panicles in the irrigation strategies were as follows: under good irrigation conditions (FI and FAF), the amylopectin content in grains was greater in general (**Figures 6A, B**), and the amylopectin content in the middle panicles of grains was greater than that in grains on the top and bottom panicles, which was highly positively related to the activity of SBE and DBE in the middle grain-filling stage (**Supplementary Figure S1B**). However, the amylose content in SAF was higher than that in the DI, FI, and FAF treatments. Finally, compared with FI, DI, and FAF, the ratio of amylose content/amylopectin content in SAF increased by 23.8% (**Figure 6C**), which affected the nutritional quality of drip-irrigated rice (Zhou et al., 2021). In a previous study demonstrating that alternating wet and dry irrigation enhances amylose and protein levels in rice grains, the quality of rice significantly decreased (Cheng et al., 2003). In contrast, the results of light and heavy alternating wet and dry irrigation were the opposite (Zhang et al., 2008; Xu et al., 2019), which shows that rice quality will undoubtedly decrease during drought in rice planting. According to Cheng et al. (2003), the main cause of the decline in the nutritional quality of rice is increased amylose and protein content, which is consistent with the results of our study. In our investigation, rice quality was affected by water supply during the grain-filling stage. However, the changes in the key enzymes of starch synthesis require further research, such as tracking the dynamics of starch synthase with water during the grain-filling period and better matching the source-sink relationship, which are important reference points for elucidating the mechanism that regulates the yield and quality of drip-irrigated rice.

## Conclusions

Post-anthesis supplementary irrigation stimulated the activities of ADP-glucose pyrophosphorylase and starch branching enzymes in the grains in the middle of panicles, which helped increase the content of amylopectin, improve sink activity, promote grain filling, and improve grain yield. This irrigation also increased the glutenin-to-total protein ratio and reduced the gliadin-to-total protein ratio. In short, post-anthesis supplementary irrigation promoted the source-sink balance of drip-irrigated rice, which is conducive to improving both grain yield and quality and maintaining high water productivity. Post-anthesis irrigation for drip-irrigated rice boosted grain yield and the harvest index by 29.2 and 11%, respectively, compared to normal drip irrigation, and increased water productivity by 19% compared with flooding irrigation.

## Data availability statement

The original contributions presented in the study are included in the article/**Supplementary Material**. Further inquiries can be directed to the corresponding author.

## Author contributions

CW and XW designed the experiments. XW, LL, GF, XL and YY performed the experiments. XW, LL and JW performed the data analysis. XW wrote the first draft of the manuscript, and CW and XZ revised the manuscript and managed the projects. All authors contributed to the article and approved the submitted version.
