# Supplementary material for: Post-anthesis supplementary irrigation improves grain yield and nutritional quality of drip-irrigated rice (Oryza sativa L.)
Source: Front Plant Sci. 2023 Apr 5;14:1126278. doi: 10.3389/fpls.2023.1126278 (PMC10113464; doi:10.3389/fpls.2023.1126278)
Supplement: Supplementary file 1 [file DataSheet_1.docx]

Supplementary Material

# Supplementary Figures and Tables

## Supplementary Figures


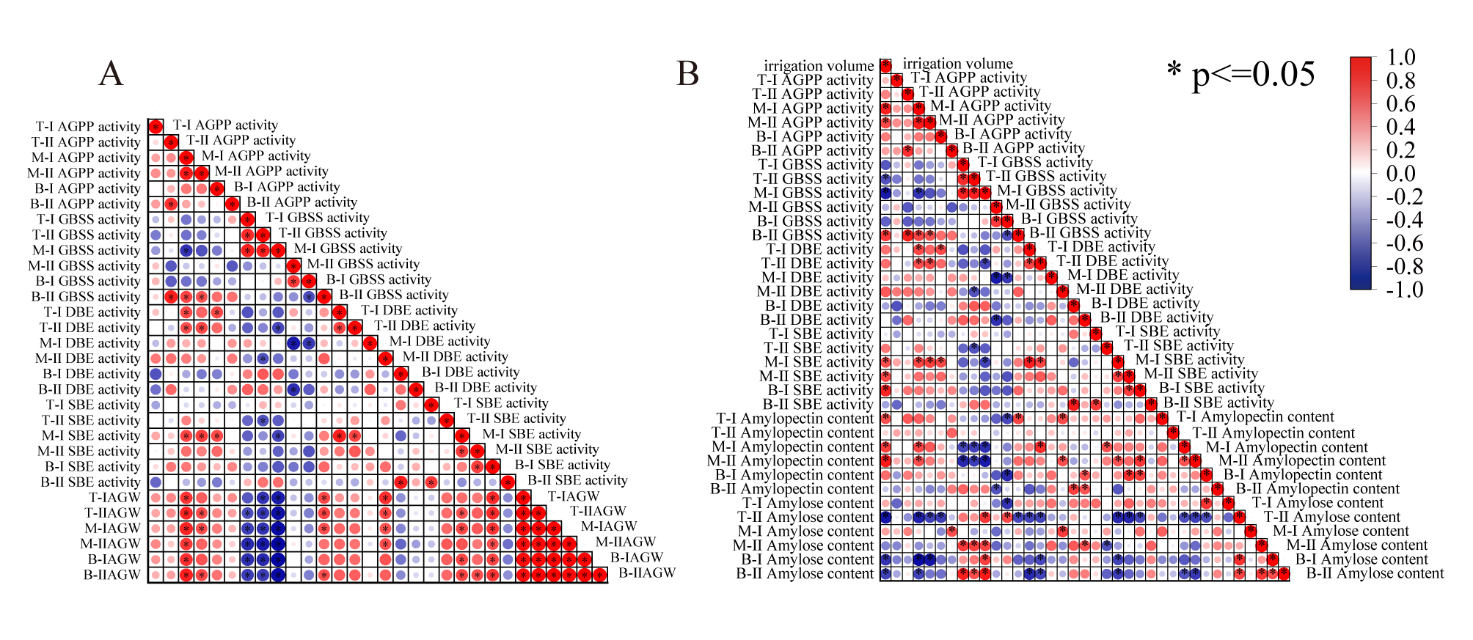


**Supplementary Figure S1.** Correlation analysis between the activities of key starch synthesis enzymes and average grain weight in grains on different positions of rice panicles (A); Correlation analysis among the activities of key enzymes for starch synthesis, amylopectin and amylopectin in grains on different positions, and irrigation amount (B), *p<0.05. Abbreviation: AWG represents average grain weight; T, M and B represent the top, middle and bottom of the panicles respectively, and the Roman numerals I and II represent the primary and secondary branches respectively.

## Supplementary Tables

Table S1 Protein content and protein composition of grains on different panicle positions under different irrigation strategy treatments.

| Irrigation strategies | Positions on panicle | w/% | | | | | |
| --- | --- | --- | --- | --- | --- | --- | --- |
|  |  | Total | Albumin | Globulin | Prolamin | Glutenin | Others |
| FI | T-Ⅰ | 8.54 d | 10.40 a | 7.56 a | 14.63 b | 35.79 c | 31.62 ab |
|  | T-Ⅱ | 9.02 cd | 8.33 b | 7.78 a | 14.89 b | 53.10 a | 15.90 c |
|  | M-Ⅰ | 10.69 a | 7.00 c | 6.76 b | 10.52 d | 37.26 c | 38.46 a |
|  | M-Ⅱ | 9.03 cd | 10.02 a | 6.62 b | 14.27 bc | 39.38 c | 29.71 ab |
|  | B-Ⅰ | 9.45 bc | 8.37 b | 6.80 b | 13.29 c | 47.15 ab | 24.39 bc |
|  | B-Ⅱ | 9.62 b | 9.77 a | 7.43 a | 17.86 a | 42.01 bc | 22.92 bc |
| DI | T-Ⅰ | 10.64 b | 7.16 de | 5.91 d | 14.31 d | 31.38 bc | 41.24 ab |
|  | T-Ⅱ | 10.70 b | 8.61 cd | 6.06 a | 17.70 c | 33.76 bc | 33.87 bc |
|  | M-Ⅰ | 11.10 a | 6.23 e | 5.77 e | 12.01 e | 26.51 c | 49.47 a |
|  | M-Ⅱ | 8.04 d | 7.75 a | 7.72 c | 19.27 b | 41.88 ab | 23.38 cd |
|  | B-Ⅰ | 9.88 c | 7.49 bc | 6.25 c | 14.10 d | 35.59 bc | 36.57 b |
|  | B-Ⅱ | 10.04 c | 8.27 b | 6.38 b | 21.00 a | 45.43 a | 18.93 d |
| SAF | T-Ⅰ | 10.06 bc | 8.29 b | 5.94 b | 14.02 d | 35.72 bc | 36.02 ab |
|  | T-Ⅱ | 9.85 cd | 6.89 c | 6.56 a | 21.12 a | 43.38 a | 22.06 c |
|  | M-Ⅰ | 9.27 d | 10.35 a | 6.84 a | 17.83 b | 40.33 ab | 24.66 c |
|  | M-Ⅱ | 10.68 ab | 7.80 b | 5.26 c | 15.99 c | 31.66 c | 39.28 a |
|  | B-Ⅰ | 10.65 ab | 6.80 c | 5.70 b | 13.21 d | 42.13 a | 32.17 b |
|  | B-Ⅱ | 11.16 a | 6.62 c | 5.17 c | 17.12 bc | 35.28 bc | 35.81 ab |
| FAF | T-Ⅰ | 7.60 b | 8.44 c | 8.78 a | 19.91 a | 46.50 a | 16.37 b |
|  | T-Ⅱ | 7.79 b | 10.45 b | 8.53 a | 20.58 a | 35.09 b | 25.34 ab |
|  | M-Ⅰ | 7.85 b | 12.23 a | 8.24 a | 15.95 bc | 46.88 a | 16.70 b |
|  | M-Ⅱ | 9.04 a | 8.25 c | 6.75 c | 15.49 c | 38.72 ab | 30.78 a |
|  | B-Ⅰ | 9.28 a | 8.08 c | 7.09 bc | 15.03 c | 46.39 a | 23.41 ab |
|  | B-Ⅱ | 9.13 a | 10.17 b | 7.54 b | 17.48 b | 37.46 b | 27.34 a |

Note: The data are mean ± standard deviation of three replicates. Different letters showed significant difference among treatments (P<0.05). The Least-Significant Difference Test (LSD) was used for comparison. Abbreviation: FI, the whole growing season flooding; DI, the whole growing season normal drip irrigation (soil relative moisture (RSM) was maintained in a range of 90-100%); SAF, pre-anthesis normal drip irrigation and post-anthesis water stress (the RSM was maintained in a range of 80-90% after anthesis); FAF, pre-anthesis normal drip irrigation and post-anthesis flooding; T, M and B represent the top, middle and bottom of the panicles respectively, and the Roman numerals I and II represent the primary and secondary branches respectively.

Table S2 Photosynthetic characteristics values under different irrigation strategy treatments.

| Irrigation strategies | Pn | Tr | Gs | Ci |
| --- | --- | --- | --- | --- |
|  | (mmolCO_2_ m^-2^ s^-1^) | (mmolH_2_Om^-2^ s^-1^) | (mmol·m^-2^ s^-1^) | (μmolCO_2_ mol^-1^) |
| FI | 11.35±2.08 a | 5.30±0.28 a | 237.50±24.53 a | 290.06±8.23 a |
| DI | 8.12+0.93b | 5.15+0.59 a | 116.38+16.08 bc | 206.07+6.83 c |
| SAF | 5.45±0.68 c | 3.90±0.29 b | 89.83±7.47 c | 214.13±7.59 c |
| FAF | 10.13±1.48 a | 5.12±0.49 a | 163.40±25.26 b | 266.47±5.40 b |

Note: The data are mean ± standard deviation of three replicates. Different letters showed significant difference among treatments (P<0.05). The Least-Significant Difference Test (LSD) was used for comparison. Abbreviation: FI, the whole growing season flooding; DI, the whole growing season normal drip irrigation (soil relative moisture (RSM) was maintained in a range of 90-100%); SAF, pre-anthesis normal drip irrigation and post-anthesis water stress (the RSM was maintained in a range of 80-90% after anthesis); FAF, pre-anthesis normal drip irrigation and post-anthesis flooding; Pn represents net photosynthetic rate; Tr represents transpiration rate; Gs represents pore conductivity; Ci represents intercellular CO_2_ concentration.
